# Radium-223 in women with hormone receptor-positive bone-metastatic breast cancer receiving endocrine therapy: pooled analysis of two international, phase 2, randomized, double-blind, placebo-controlled trials

**DOI:** 10.1007/s10549-023-07147-z

**Published:** 2023-12-20

**Authors:** Hope S. Rugo, Catherine H. Van Poznak, Patrick Neven, Iwona Danielewicz, Soo Chin Lee, Mario Campone, Jeannie Y. K. Chik, Estela Vega Alonso, Bjørn Naume, Etienne Brain, Jonathan M. Siegel, Rui Li, Deise Uema, Volker J. Wagner, Robert E. Coleman

**Affiliations:** 1 University of California San Francisco Helen Diller Family Comprehensive Cancer Center, San Francisco, CA USA; 2 University of Michigan Rogel Cancer Center, Ann Arbor, MI USA; 3 University Hospitals Leuven, Louvain, Belgium; 4 Szpital Morski Im. PCK, Gdynia, Poland; 5 National University Hospital (S) Pte Ltd, Singapore, Singapore; 6 Institut de Cancerologie de l’Ouest, St Herblain, France; 7 Queen Elizabeth Hospital, Kowloon, Hong Kong; 8 Centro Integral Oncológico Clara Campal, Madrid, Spain; 9 Institute of Clinical Medicine, University of Oslo, and Oslo University Hospital, Oslo, Norway; 10 Institut Curie – René-Huguenin Hospital, Saint-Cloud, France; 11 Bayer HealthCare Pharmaceuticals Inc., Whippany, NJ USA; 12 Bayer Consumer Care AG, Basel, Switzerland; 13 Cancer Clinical Trials Centre, University of Sheffield, Weston Park Hospital, Sheffield, UK; 14 Department of Medicine (Hematology/Oncology), University of California San Francisco Helen Diller Family Comprehensive Cancer Center, 1825 4th St., 3rd Floor, San Francisco, CA 94158 USA; 15 Department of Oncology and Metabolism, Cancer Clinical Trials Centre, Weston Park Hospital, Broomcross Building, Floor 2, Whitham Road, Sheffield, S10 2SJ UK

**Keywords:** Bone metastasis, Breast cancer, Hormone receptor positive, Endocrine therapy, Radium-223, Symptomatic skeletal event-free survival

## Abstract

**Background:**

Most women with advanced breast cancer have skeletal metastases. Radium-223 is an alpha-emitting radionuclide that selectively targets areas of bone metastases.

**Methods:**

Two double-blind, placebo-controlled studies of radium-223 were conducted in women with hormone receptor-positive (HR+), bone-predominant metastatic breast cancer. All patients received endocrine therapy (ET), as a single agent of the investigator’s choice (Study A) or exemestane + everolimus (Study B). Patients were randomized to receive radium-223 (55 kBq/kg) or placebo intravenously every 4 weeks for six doses. Accrual was halted following unblinded interim analyses per protocol amendments, and both studies were terminated. We report pooled analyses of symptomatic skeletal event-free survival (SSE-FS; primary endpoint), radiologic progression-free survival (rPFS) and overall survival (OS; secondary), and time to bone alkaline phosphatase (ALP) progression (exploratory).

**Results:**

In total, 382 patients were enrolled, and 196 SSE-FS events (70% planned total) were recorded. Hazard ratios (95% confidence intervals) and nominal *p* values for radium-223 + ET versus placebo + ET were: SSE-FS 0.809 (0.610–1.072), *p* = 0.1389; rPFS 0.956 (0.759–1.205), *p* = 0.7039; OS 0.889 (0.660–1.199), *p* = 0.4410; and time to bone ALP progression 0.593 (0.379–0.926), *p* = 0.0195. Radium-223- or placebo-related treatment-emergent adverse events were reported in 50.3% versus 35.1% of patients (grade 3/4: 25.7% vs. 8.5%), with fractures/bone-associated events in 23.5% versus 23.9%.

**Conclusions:**

In patients with HR+ bone-metastatic breast cancer, numeric differences favoring radium-223 + ET over placebo + ET for the primary SSE-FS endpoint were suggestive of efficacy, in line with the primary outcome measure used in the underlying phase 2 studies. No similar evidence of efficacy was observed for secondary progression or survival endpoints. Adverse events were more frequent with radium-223 + ET versus placebo + ET, but the safety profile of the combination was consistent with the safety profiles of the component drugs.

*Clinical trial registration numbers*

Study A: NCT02258464, registered October 7, 2014.

Study B: NCT02258451, registered October 7, 2014.

**Supplementary Information:**

The online version contains supplementary material available at 10.1007/s10549-023-07147-z.

## Introduction

Approximately 70% of patients with advanced breast cancer have skeletal metastases [1]. These patients are at risk of increased morbidity, including skeletal-related events (e.g., bone pain requiring radiotherapy or surgery, pathologic fractures, spinal cord compression, and symptomatic hypercalcemia), which impair health-related quality of life and functional independence [1, 2].

Radium-223 is an alpha-emitting radionuclide that acts as a calcium mimetic and is preferentially taken up at sites of increased osteoblastic activity associated with bone metastases. The high-energy, short-range alpha particles produced by radium-223 cause irreversible double-strand DNA breaks and subsequent tumor cell death [3]. In the ALSYMPCA trial, which was conducted in men with metastatic castration-resistant prostate cancer and symptomatic bone metastases, radium-223 significantly improved overall survival (OS), delayed symptomatic skeletal complications, and improved or maintained health-related quality of life (exploratory analysis) compared with placebo, and was well tolerated [4–6].

Given the efficacy shown in the ALSYMPCA trial, radium-223 may be an appropriate intervention for evaluation in other cancers associated with bone metastases, including hormone receptor-positive (HR+) breast cancer [1, 7–12], for which few therapeutic options specifically targeting bone lesions currently exist [9]. Early-phase clinical studies in breast cancer indicated that radium-223 had antitumor activity, particularly in patients with bone-only metastases. In a single-arm phase 2 study of 36 patients with HR+, bone-dominant metastatic breast cancer, the disease control rate at 9 months was 49% and the tumor response rate at 6 months was 54%, with a median progression-free survival of 7.4 months overall and 13.8 months in patients with bone-only metastases [7]. In a separate single-arm phase 2a study of 23 patients with advanced breast cancer and progressive, bone-dominant disease, who were no longer candidates for further endocrine therapy, radium-223 was shown to target areas of increased bone metabolism caused by metastases: bone markers such as urinary N-telopeptide type 1 and serum bone alkaline phosphatase were consistently reduced over 16 weeks of radium-223 treatment (four doses), and 50 of 155 hypermetabolic lesions on fluorine-18-fluorodeoxyglucose positron emission tomography–computed tomography (CT) showed metabolic decrease after two doses of radium-223 [10]. Other smaller studies and case reports have also indicated tolerability and promising signs of efficacy in patients with bone-dominant advanced breast cancer treated with radium-223.

Following these encouraging results, two randomized phase 2 studies were conducted, in which patients were randomized to receive radium-223 + endocrine therapy or placebo + endocrine therapy. In Study A, patients received a single agent of the investigator’s choice as background endocrine therapy. In Study B, patients received study-directed exemestane + everolimus as a component of the combination therapy under investigation. Key results for these studies have been reported in congress presentations [13, 14] and are available at ClinicalTrials.gov but have not been published separately. Here, we present full results from the two studies, as a pooled analysis of efficacy and safety data.

## Methods

### Study design and participants

Study A (ClinicalTrials.gov identifier: NCT02258464) and Study B (NCT02258451) were international phase 2, randomized, double-blind, placebo-controlled, parallel-group trials. The study protocols and all protocol amendments were reviewed and approved by each study site’s independent ethics committee or institutional review board. The studies were conducted in accordance with all local legal and regulatory requirements and with the principles in the Declaration of Helsinki and the International Council for Harmonisation guideline E6: Good Clinical Practice. All patients signed an informed consent form before participation. The full protocols are available at ClinicalTrials.gov.

In both studies, inclusion criteria included age at least 18 years; HR+ and human epidermal growth factor receptor 2-negative (HER2−) breast cancer not amenable to curative treatment with surgery or radiotherapy; bone-dominant disease with at least two skeletal metastases identified at baseline by bone scintigraphy and confirmed by CT or magnetic resonance imaging (MRI); and one or more prior lines of hormonal therapy in the metastatic setting. Soft tissue and/or visceral metastases were allowed, as long as the patient was not being considered for treatment with chemotherapy for immediately life-threatening visceral disease. Both studies allowed the inclusion of postmenopausal and premenopausal patients; however, in Study B, premenopausal patients had to be under ovarian suppression (ovarian radiation or concomitant therapy with a luteinizing hormone-releasing hormone agonist/antagonist). In Study A, patients had to be eligible for treatment with selective estrogen receptor modulators, steroidal or nonsteroidal aromatase inhibitors, or fulvestrant as a second or later line of therapy. In Study B, they had to have received prior letrozole or anastrozole in an adjuvant or metastatic setting and be eligible for exemestane + everolimus as a second or later line of therapy; patients receiving everolimus before study entry were ineligible. Prior chemotherapy for metastatic disease and prior systemic or hemibody external radiotherapy were not allowed in either study. Prior palliative radiotherapy to bone was permitted. Full inclusion and exclusion criteria for each study are listed in the Supplementary Methods.

### Study interventions

In both studies, patients were randomized 1:1 in a double-blind fashion to receive radium-223 + endocrine therapy or placebo + endocrine therapy. Randomization was stratified by geographic region (Europe [including Israel] and North America vs. Asia; both studies]; previous lines of endocrine therapy in the metastatic setting (1 vs. ≥ 2; both studies); and prior skeletal-related events (1 vs. 2; Study A) or visceral metastases (present vs. absent; Study B). Full randomization and blinding details can be found in the Supplementary Methods. Radium-223 dichloride 55 kBq/kg body weight or placebo (0.9% sodium chloride solution for injection) was administered intravenously as a slow bolus injection for a maximum of six cycles at intervals of 4 weeks. Details of study drug storage and handling, and dose calculation, calibration, preparation, administration, and adjustments, are provided in the Supplementary Methods.

In Study A, all patients received the investigator’s choice of endocrine therapy with single-agent tamoxifen, anastrozole, letrozole, fulvestrant, or exemestane as background therapy. Endocrine therapy was administered according to standard local practice and was started within 15 days before randomization and no later than the first radium-223 or placebo injection; it could continue after completion of study treatment with radium-223 or placebo.

In Study B, study treatment included exemestane (25 mg once daily recommended) and everolimus (10 mg once daily recommended), starting after randomization but before or with the first radium-223 or placebo injection. Endocrine-based treatment continued until disease progression or premature discontinuation due to one or more of the following: unacceptable toxicity, inability to attend clinic visits, initiation of prohibited therapy, treatment delay for more than 4 weeks, or withdrawal of consent.

In both studies, all participants had to have been receiving bone health agents (bisphosphonates or denosumab) for at least 1 month before the start of study intervention and then be maintained on these agents throughout the study.

### Assessments

In the individual studies and the pooled analysis, the primary endpoint was symptomatic skeletal event (SSE)-free survival (SSE-FS), defined as the time from randomization to the first occurrence of an on-study SSE or death from any cause. SSEs were defined as use of external-beam radiation therapy to relieve skeletal symptoms, new symptomatic pathologic bone fractures (vertebral or nonvertebral), spinal cord compression, or a tumor-related orthopedic surgical intervention. In Study A, bone scans and CT/MRI were scheduled every 12 weeks; in Study B, bone scans were scheduled every 12 weeks and CT/MRI every 8 weeks. In this pooled analysis, OS and radiologic progression-free survival (rPFS) were secondary endpoints, and time to bone alkaline phosphatase (ALP) progression was an exploratory endpoint.

Adverse events were recorded during the study treatment period (from start of first study treatment to 30 days after last study treatment, including the endocrine-based regimen in Study B). Febrile neutropenia and hemorrhage events were recorded during active follow-up. Adverse events in the pooled analysis are coded according to the Medical Dictionary for Regulatory Activities version 23.1; earlier versions were used in the original studies. Adverse event severity is documented using National Cancer Institute Common Terminology Criteria for Adverse Events version 4.03. Safety variables and follow-up details are defined in the Supplementary Methods.

### Sample size

In each study, the sample size was planned to allow analysis of the primary efficacy endpoint, SSE-FS, using a log-rank test stratified by study randomization factors, with a two-sided alpha of 0.2, power of 90%, and randomization ratio of 1:1. In Study A, 227 patients and 119 events were required to detect a hazard ratio (HR) of 0.625. In Study B, 311 patients and 160 events were required to detect a HR of 0.667. No sample size calculation was made for the pooled analysis.

In both studies, enrollment was slower than expected, as the treatment landscape evolved to incorporate new targeted treatment options [15, 16]. Accrual was halted for an unblinded interim analyses of efficacy and safety as authorized by protocol amendments. Once the interim analysis was completed, however, the decision was taken not to resume enrollment (see the Supplementary Methods for details). In Study A, 44% of the planned population was enrolled (99/227), and 39% of the planned SSEs occurred (47/119). In Study B, 91% of the planned population was enrolled (283/311), and 93% of the planned events occurred (149/160).

The pooled descriptive analysis includes 71% of the planned patients (382/538) in the intention-to-treat (ITT) population and 70% (375/538) in the safety population. A total of 70% of the 279 pooled planned events occurred. Although no power calculation was performed, the 196 pooled events were greater than the 160 events planned for Study B, which was designed for development decision-making purposes, with 90% power to detect a 50% increase in SSE-FS with a 0.10 one-sided significance level (0.20 two-sided). The liberal significance level is appropriate for a phase 2 study.

### Statistical methods

Statistical analysis was performed using SAS version 9.4 (SAS Institute Inc., Cary, NC, USA). In this pooled analysis, the findings are reported descriptively. There is no alpha adjustment, and all *p*-values provided are two-sided, nominal, and for descriptive purposes only. In general, number, mean (standard deviation), and/or median (range) are provided for numerical data, and frequency tables for categorical data.

All efficacy analyses are based on the ITT analysis set, which comprises all randomized patients according to the treatment assigned at randomization. For time-to-event endpoints, Kaplan–Meier curves are plotted and medians with Brookmeier–Crowley confidence intervals (CIs) calculated for each arm. Differences between treatment arms are reported as HRs with 95% CIs using univariate Cox models stratified by study (A vs. B). Pooled analysis log-rank tests are stratified by study (A vs. B) and are provided for descriptive purposes only.

The safety analysis set comprises all randomized patients who received at least one dose of radium-223 or placebo and is analyzed according to treatment actually received, with descriptive statistics for all safety analyses.

## Results

### Patient disposition and baseline characteristics

Of 540 patients screened in the two studies, 382 were randomized to receive radium-223 + endocrine therapy (*n* = 191) or placebo + endocrine therapy (*n* = 191). Seven patients (radium-223 + endocrine therapy *n* = 3; placebo + endocrine therapy *n* = 4) did not start their assigned treatment, and 304 patients (radium-223 + endocrine therapy *n* = 153; placebo + endocrine therapy *n* = 151) discontinued study treatment (including exemestane + everolimus in Study B) (Supplementary Fig. 1). The most common reasons for discontinuation of study treatment were disease progression (radium-223 + endocrine therapy *n* = 103 [54%]; placebo + endocrine therapy *n* = 119 [62%]) and patient decision (*n* = 26 [14%] and *n* = 16 [8%], respectively). In total, 105 patients (56.1%) completed six cycles of radium-223, and 101 (53.7%) completed six cycles of placebo.

Demographic and baseline characteristics are shown in Table 1 for the combined population and in Supplementary Tables 1 and 2 for the individual studies. The two treatment arms were generally balanced, with small differences in progesterone receptor-positive status and median times from initial diagnosis to bone metastases and to randomization (all slightly higher with radium-223 + endocrine therapy vs. placebo + endocrine therapy).

**Table 1:** Demographic and baseline characteristics

Patient demographics and baseline characteristics (intention-to-treat population, *N* = 382)	Radium-223 + endocrine therapy (*n* = 191)	Placebo + endocrine therapy (*n* = 191)
Mean age at baseline, years (*SD*)	59.2 (10.7)	59.0 (11.7)
Age group, *n* (%)		
< 55 years	63 (33.0)	70 (36.6)
≥ 55 years	128 (67.0)	121 (63.4)
ECOG performance status at baseline, *n* (%)		
Missing data	0	3 (1.6)
0	89 (46.6)	89 (46.6)
1	102 (53.4)	99 (51.8)
Menopausal status at baseline, *n* (%)		
Premenopausal	17 (8.9)	18 (9.4)
Postmenopausal	174 (91.1)	173 (90.6)
Progesterone receptor status at initial diagnosis, *n* (%)		
Positive	160 (83.8)	141 (73.8)
Negative	26 (13.6)	40 (20.9)
Unknown	5 (2.6)	10 (5.2)
Previous lines of endocrine therapy in metastatic setting, *n* (%)		
1	107 (56.0)	107 (56.0)
≥ 2	84 (44.0)	84 (44.0)
Metastatic status at baseline, *n* (%)		
Bone metastases only	90 (47.1)	89 (46.6)
Bone plus visceral metastases	72 (37.7)	76 (39.8)
Bone plus non-visceral metastases	29 (15.2)	26 (13.6)
Prior SREs, *n* (%)		
1	130 (68.1)	125 (65.4)
2	61 (31.9)	64 (33.5)
Prior palliative radiotherapy to bone, *n* (%)		
0	186 (97.4)	180 (94.2)
1	4 (2.1)	10 (5.2)
2	1 (0.5)	1 (0.5)

*ECOG* Eastern Cooperative Oncology Group, *SD* standard deviation, *SRE* skeletal-related event

### Primary efficacy endpoint: SSE-FS (ITT)

**Fig. 1:**
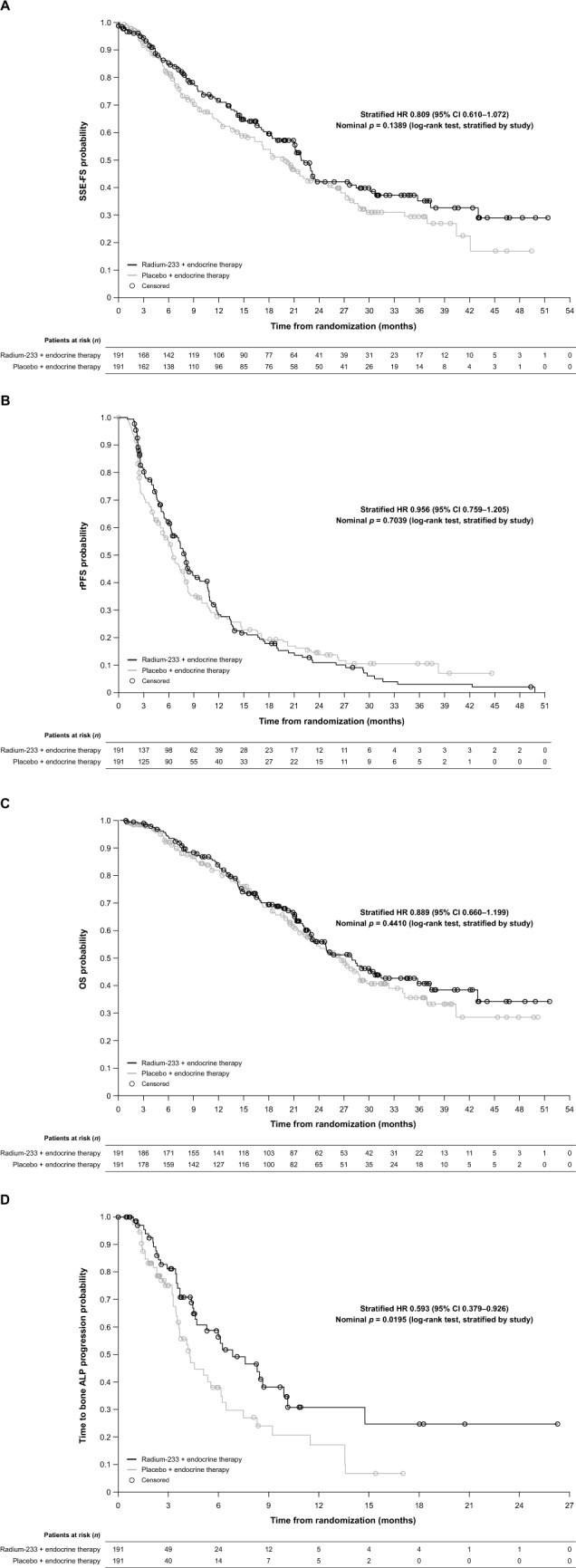
Kaplan–Meier estimates of efficacy endpoints **a** Symptomatic skeletal event-free survival (SSE-FS) (primary endpoint), **b** radiographic progression-free survival (rPFS) (secondary endpoint), **c** overall survival (OS) (secondary endpoint), and **d** time to bone alkaline phosphatase (ALP) progression (exploratory endpoint): intention-to-treat population. Missing bone ALP measurements resulted in high patient censoring. *CI* confidence interval, *HR* hazard ratio

By the data cut-offs of August 13, 2019, for Study A and January 22, 2020, for Study B, a total of 196 SSE-FS events had occurred. In the pooled analysis, the observed median SSE-FS was 22.2 months in the radium-223 + endocrine arm and 19.9 months in the placebo + endocrine therapy arm. The HR was 0.809 (95% CI 0.610–1.072; *p* = 0.1389) for the radium-223 + endocrine therapy arm versus the placebo + endocrine therapy arm (Table 2; Fig. 1a). SSE-FS outcomes for the individual studies are presented in Supplementary Table 3.

**Table 2:** Efficacy outcomes (intention-to-treat population, *N* = 382)

Endpoint	Radium-223 + endocrine therapy (*n* = 191)		Placebo + endocrine therapy (*n* = 191)		HR^a^ (95% CI)	*p*-value^b^
	Patients with event, *n*	Median time to event, months (95% CI)	Patients with event, *n*	Median time to event, months (95% CI)		
SSE-FS (primary)	90	22.2 (18.4–27.6)	106	19.9 (16.2–24.2)	0.809 (0.610–1.072)	0.1389
OS (secondary)	85	28.0 (23.2–35.8)	89	26.4 (22.6–29.7)	0.889 (0.660–1.199)	0.4410
rPFS (secondary)	146	7.9 (6.4–9.2)	145	6.5 (5.3–7.9)	0.956 (0.759–1.205)	0.7039
Time to bone ALP progression (exploratory)^c^	35	6.9 (4.6–9.9)	45	4.3 (3.6–6.1)	0.593 (0.379–0.926)	0.0195

*ALP* alkaline phosphatase, *CI* confidence interval, *HR* hazard ratio, *OS* overall survival, *rPFS* radiologic progression-free survival, *SSE-FS* symptomatic skeletal event-free survival

^a^Stratified as outlined in *Methods*

^b^Two-sided log-rank test

^c^61 patients (62%) in Study A and 165 patients (58%) in Study B were censored at randomization because of missing bone ALP assessment at baseline and/or post-baseline

### Secondary and exploratory efficacy endpoints (ITT)

The HR for rPFS was 0.956 (95% CI 0.759–1.205; *p* = 0.7039), and the HR for OS was 0.889 (95% CI 0.660–1.199; *p* = 0.4410) (Table 2; Fig. 1b and c). No difference was observed for rPFS or OS between radium-223 + endocrine therapy and placebo + endocrine therapy arms. The first rPFS event was bone progression in 42 patients (28.8%) who received radium-223 + endocrine therapy and 53 patients (36.6%) who received placebo + endocrine therapy, non-bone progression in 99 (67.8%) and 86 (59.3%), respectively, and death in 5 (3.4%) and 6 (4.1%), respectively.

In patients with bone ALP measurements, the HR for time to bone ALP progression was 0.593 (95% CI 0.379–0.926; *p* = 0.0195) for radium-223 + endocrine therapy versus placebo + endocrine therapy (Table 2; Fig. 1d).

Secondary and exploratory efficacy outcomes for the individual studies are presented in Supplementary Table 3.

### Safety

Generally, safety outcomes were similar between the radium-223 + endocrine therapy and placebo + endocrine therapy arms (Tables 3 and 4 for the combined population and Supplementary Tables 4 and 5 for the individual studies). However, there was a greater incidence of radium-223- or placebo-related treatment-emergent adverse events (TEAEs) of any grade (50.3% vs. 35.1%) and grade 3 or 4 (25.7% vs. 8.5%) in the radium-223 + endocrine therapy versus placebo + endocrine therapy arm. As anticipated, there were more TEAEs in factors associated with bone marrow reserve and fewer TEAEs of back pain and bone pain.

**Table 3:** Treatment-emergent adverse events (safety population, *N* = 375)

Patients with TEAEs, *n* (%)	Radium-223 + endocrine therapy (*n* = 187)	Placebo + endocrine therapy (*n* = 188)
Any TEAE	185 (98.9)	182 (96.8)
Grade 1/2	64 (34.2)	74 (39.4)
Grade 3/4	113 (60.4)	100 (53.2)
Grade 5^a^	8 (4.3)	8 (4.3)
Serious	60 (32.1)	65 (34.6)
Leading to dose modification	27 (14.4)	18 (9.6)
Leading to discontinuation	13 (7.0)	10 (5.3)
Radium-223-/placebo-related TEAEs	94 (50.3)	66 (35.1)
Grade 1/2	46 (24.6)	50 (26.6)
Grade 3/4	48 (25.7)	16 (8.5)
Grade 5	0	0
Serious	11 (5.9)	5 (2.7)
Leading to dose modification	15 (8.0)	7 (3.7)
Leading to discontinuation	6 (3.2)	3 (1.6)

Adverse event severity is graded according to National Cancer Institute Common Terminology Criteria for Adverse Events version 4.03

*TEAE* treatment-emergent adverse event

^a^Grade 5 adverse events that occurred during treatment (between the first dose of study drug and up to 30 days after the last dose of study drug; *n* = 13) or active follow-up (> 30 days after the last dose of study drug; *n* = 3) were recorded as follows: Study A radium-223 + endocrine therapy arm: no events; placebo + endocrine therapy arm: seizure associated with clinical disease progression (*n* = 1); Study B radium-223 + endocrine therapy arm: general physical health deterioration (*n* = 2), metastases to meninges (*n* = 2), pneumonia (*n* = 1), pulmonary embolism (*n* = 1, possibly related to everolimus treatment), respiratory failure (*n* = 1), and sudden death (*n* = 1); placebo + endocrine therapy arm: general physical health deterioration (*n* = 1), bone pain (*n* = 1), death (not otherwise specified, *n* = 1), malaise (*n* = 1), multiple organ dysfunction syndrome (*n* = 1), performance status decreased (*n* = 1), and subileus (*n* = 1)

**Table 4:** TEAEs occurring in ≥ 10% of patients and treatment-related TEAEs occurring in ≥ 5% of patients in either treatment arm (safety population, *N* = 375)

Patients with TEAEs, *n* (%)	Radium-223 + endocrine therapy (*n* = 187)		Placebo + endocrine therapy (*n* = 188)	
	Any grade	Grade ≥ 3	Any grade	Grade ≥ 3
Nonhematologic TEAEs				
Stomatitis	68 (36.4)	8 (4.3)	69 (36.7)	8 (4.3)
Nausea	53 (28.3)	1 (0.5)	39 (20.7)	1 (0.5)
Diarrhea	53 (28.3)	8 (4.3)	38 (20.2)	2 (1.1)
Decreased appetite	51 (27.3)	2 (1.1)	39 (20.7)	3 (1.6)
Fatigue	50 (26.7)	7 (3.7)	50 (26.6)	4 (2.1)
Arthralgia	45 (24.1)	2 (1.1)	56 (29.8)	5 (2.7)
Headache	37 (19.8)	0	33 (17.6)	0
Vomiting	32 (17.1)	1 (0.5)	31 (16.5)	2 (1.1)
Weight decreased	31 (16.6)	1 (0.5)	24 (12.8)	1 (0.5)
Asthenia	30 (16.0)	1 (0.5)	27 (14.4)	4 (2.1)
Peripheral edema	29 (15.5)	0	26 (13.8)	1 (0.5)
Cough	26 (13.9)	0	30 (16.0)	0
Pain in extremity	26 (13.9)	1 (0.5)	25 (13.3)	3 (1.6)
Back pain	25 (13.4)	1 (0.5)	34 (18.1)	4 (2.1)
Pyrexia	23 (12.3)	0	17 (9.0)	1 (0.5)
Alanine aminotransferase increased	22 (11.8)	10 (5.3)	25 (13.3)	8 (4.3)
Bone pain	22 (11.8)	7 (3.7)	29 (15.4)	8 (4.3)
Rash	21 (11.2)	1 (0.5)	31 (16.5)	0
Hypokalemia	20 (10.7)	10 (5.3)	15 (8.0)	10 (5.3)
Pneumonitis	19 (10.2)	5 (2.7)	19 (10.1)	2 (1.1)
Dyspnea	19 (10.2)	1 (0.5)	18 (9.6)	3 (1.6)
Aspartate aminotransferase increased	18 (9.6)	8 (4.3)	22 (11.7)	5 (2.7)
Upper respiratory tract infection	18 (9.6)	0	21 (11.2)	0
Constipation	10 (5.3)	0	24 (12.8)	0
Hematologic TEAEs				
Anemia	56 (29.9)	34 (18.2)	44 (23.4)	23 (12.2)
Neutropenia	34 (18.2)	18 (9.6)	11 (5.9)	1 (0.5)
Thrombocytopenia	24 (12.8)	6 (3.2)	10 (5.3)	3 (1.6)
Drug-related TEAEs				
Nonhematologic				
Fatigue	23 (12.3)	5 (2.7)	10 (5.3)	1 (0.5)
Nausea	18 (9.6)	0	13 (6.9)	1 (0.5)
Diarrhea	13 (7.0)	1 (0.5)	8 (4.3)	0
Vomiting	5 (2.7)	0	11 (5.9)	1 (0.5)
Hematologic				
Anemia	31 (16.6)	21 (11.2)	18 (9.6)	11 (5.9)
Neutropenia	23 (12.3)	11 (5.9)	4 (2.1)	0
Thrombocytopenia	12 (6.4)	3 (1.6)	3 (1.6)	1 (0.5)
Leukopenia	11 (5.9)	6 (3.2)	1 (0.5)	0

A patient is counted only once within each preferred term. A patient may be counted for more than one preferred term

Adverse events are defined according to Medical Dictionary for Regulatory Activities version 23.1, and their severity is graded according to National Cancer Institute Common Terminology Criteria for Adverse Events version 4.03

*TEAE* treatment-emergent adverse event

In total, 169 patients died: 13 during radium-223/placebo treatment in Study A or any study treatment in Study B: radium-223 + endocrine therapy arm *n* = 8 (4.3%); placebo + endocrine therapy arm *n* = 5 (2.7%); and 156 during active follow-up: *n* = 76 (40.6%) and *n* = 80 (42.6%), respectively. The causes of death were generally similar between the treatment arms and were mainly due to disease progression: radium-223 + endocrine therapy arm *n* = 67 (35.8%); placebo + endocrine therapy arm *n* = 67 (35.6%). Sixteen patients (radium-223 + endocrine therapy arm *n* = 8; placebo + endocrine therapy arm *n* = 8, Study A *n* = 1; Study B *n* = 15) had grade 5 TEAEs. In 13 patients (radium-223 + endocrine therapy arm *n* = 8; placebo + endocrine therapy arm *n* = 5), the grade 5 TEAEs occurred between the first dose of study drug and up to 30 days after the last dose of study drug. In three patients (all in the placebo + endocrine therapy arm of Study B), the grade 5 TEAE started during treatment but became fatal during follow-up (> 30 days after last dose of study drug). No grade 5 TEAEs were deemed to be related to radium-223 treatment. The overall incidence of grade 5 TEAEs in Study B was 5.4%, but none of these TEAEs was considered related to radium-223, placebo, or exemestane; one grade 5 TEAE (pulmonary embolism) was considered related to everolimus.

Bone fractures or bone-associated events were recorded in 44 patients (23.5%) treated with radium-223 + endocrine therapy and 45 patients (23.9%) treated with placebo + endocrine therapy. Pathologic fractures occurred in 10 patients (5.3%) and 12 patients (6.4%), respectively. Bone pain TEAEs were reported in 22 patients (11.8%) and 29 patients (15.4%), respectively.

## Discussion

A descriptive pooled analysis of two phase 2 studies of radium-223 + endocrine therapy in breast cancer was undertaken, because both of the underlying studies were terminated prematurely and did not reach their planned study power. Thus, the pooled analysis was based on 71% of planned patients and 70% of planned SSE-FS events. Although *p*-values were not intended for inference purposes, numeric differences favoring radium-223 + endocrine therapy over placebo + endocrine therapy for bone-related endpoints (SSE-FS and bone ALP progression) were seen in this pooled analysis, which would have reached statistical significance according to the liberal phase 2 operating criteria prespecified for the individual studies. Although correspondingly liberal CIs were not produced, descriptive 95% CIs overlapped 1 for hazard ratios. For progression and survival endpoints, 95% CIs also overlapped, and descriptive *p*-values were not suggestive of efficacy. Adverse events were more frequent in patients treated with radium-223 + endocrine therapy than in those who received placebo + endocrine therapy. It is not known whether these results would have differed if the studies had met accrual.

The impact of radium-223 + endocrine therapy on disease-related endpoints in patients with metastatic breast cancer does not appear to mirror the benefits of radium-223 seen in patients with metastatic prostate cancer in the ALSYMPCA study [4], possibly because outcomes in prostate cancer are more bone driven than in breast cancer. In the CARBON study of radium-223 in combination with capecitabine, similar results were reported, with no substantial benefit from the combination over capecitabine alone observed [17]. However, case reports indicate that radium-223 may have efficacy in patients with bone-only metastases [18], and most patients with prostate cancer have bone-only or bone-dominant metastases. Overall outcome in most solid tumors other than prostate cancer is usually driven by visceral disease, not by bone disease. Thus, the lack of efficacy of radium-223 in the clinical trials analyzed here may relate to the higher rates of visceral disease in patients with breast cancer. In our studies, although participants had bone-dominant disease and we saw signs of benefit on bone-related endpoints, visceral metastases were present in nearly 40% of participants at baseline, and most patients with rPFS events experienced progression in non-bone sites. Sites of tumor burden can affect survival, as shown in a retrospective database study of 18,322 women with metastatic breast cancer, in which the median survival from initial metastasis diagnosis was 36 months for patients with bone metastasis only, compared with 26 months for patients with metastases at any site (bone, lung, liver, brain, other, and multiple) [19].

The treatment landscape for patients with advanced HR+ HER2− breast cancer has changed considerably in recent years. In addition to endocrine therapy, the range of targeted treatment options now includes inhibitors of cell signaling pathways (e.g., cyclin dependent kinase 4/6, phosphatidylinositol-4,5-bisphosphate 3-kinase catalytic subunit alpha, or mammalian target of rapamycin inhibitors) and, in patients with *BRCA* mutations, DNA damage repair pathways (e.g., poly-ADP-ribose polymerase inhibitors) [15, 16]. As a result of the change in standard of care, the decision was taken to terminate the two studies of radium-223 + endocrine therapy. Nevertheless, there remains interest in radiopharmaceutical strategies in breast cancer. For example, a first-in-human study is in progress to assess the alpha emitter thorium-227 conjugated with a HER2 antibody, with the aim of delivering targeted therapy to HER2-expressing tumors, including breast cancer (ClinicalTrials.gov identifier NCT04147819). Another phase 1/2 study is under way in patients with triple-negative or HER2-negative breast cancer to assess a conjugate of the alpha emitter actinium-225 with an insulin-like growth factor-1 receptor-targeting humanized monoclonal antibody (ClinicalTrials.gov identifier NCT03746431). Combining a radiopharmaceutical with a non-bone-targeting agent may provide antitumor activity against both bone and visceral metastases. Studies are also planned to assess lutetium-177-labeled radiopharmaceuticals (ClinicalTrials.gov identifiers NCT04469127 and NCT04529044).

Limitations of our analysis include differences in study design between the two constituent studies, including different endocrine regimens, and variations in follow-up duration. These studies also faced missing ALP measurements and a high rate of data censoring. After enrollment was halted, some patients who had discontinued treatment were immediately transferred to a long-term follow-up study, while patients on treatment continued to be followed within Study A or Study B and thus their data remained available for the pooled analysis; therefore, some selection bias and informative censoring may have occurred. Because enrollment was stopped early in both studies, sample sizes were smaller than planned for adequate power, and the number of SSE-FS events was substantially lower than anticipated. The underlying studies had liberal operating criteria, appropriate for phase 2 trials, and this pooled analysis did not adjust for multiple comparisons. Accordingly, the pooled results should be considered descriptive only.

## Conclusion

In women with HR+ metastatic breast cancer, this pooled analysis showed a numeric improvement in control of bone disease with radium-223 + endocrine therapy over placebo + endocrine therapy, based on bone-related endpoints, with no major safety concerns. In line with the prespecified statistical analyses in the underlying phase 2 studies, which were designed to support development decisions and not to confirm efficacy or safety, the primary SSE-FS endpoint (*p* = 0.1389 two-sided) in the pooled analysis, while equivocal, could be described as showing a positive numeric trend favoring further investigation. For rPFS and OS, similar efficacy was seen between radium-223 + endocrine therapy and placebo + endocrine therapy.

### Supplementary Information

Below is the link to the electronic supplementary material.

Supplementary file1 (DOCX 118 KB)

## Data Availability

Availability of the data underlying this publication will be determined according to Bayer’s commitment to the EFPIA/PhRMA “Principles for responsible clinical trial data sharing”. This pertains to scope, timepoint and process of data access. As such, Bayer commits to sharing upon request from qualified scientific and medical researchers patient-level clinical trial data, study-level clinical trial data, and protocols from clinical trials in patients for medicines and indications approved in the United States (US) and European Union (EU) as necessary for conducting legitimate research. This applies to data on new medicines and indications that have been approved by the EU and US regulatory agencies on or after January 01, 2014. Interested researchers can use www.vivli.org to request access to anonymized patient-level data and supporting documents from clinical studies to conduct further research that can help advance medical science or improve patient care. Information on the Bayer criteria for listing studies and other relevant information is provided in the member section of the portal. Data access will be granted to anonymized patient-level data, protocols and clinical study reports after approval by an independent scientific review panel. Bayer is not involved in the decisions made by the independent review panel. Bayer will take all necessary measures to ensure that patient privacy is safeguarded.

## Abbreviations

ALP: Alkaline phosphatase
CI: Confidence interval
CT: Computed tomography
HER2−: Human epidermal growth factor receptor negative
HR: Hazard ratio
HR+: Hormone receptor positive
ITT: Intention-to-treat
MR: Magnetic resonance imaging
OS: Overall survival
rPFS: Radiologic progression-free survival
SSE: Symptomatic skeletal event
SSE-FS: Symptomatic skeletal event-free survival
TEAE: Treatment-emergent adverse event
