# Supplementary material for: Radium-223 in women with hormone receptor-positive bone-metastatic breast cancer receiving endocrine therapy: pooled analysis of two international, phase 2, randomized, double-blind, placebo-controlled trials
Source: Breast Cancer Res Treat. 2023 Dec 20;204(2):249–59. doi: 10.1007/s10549-023-07147-z (PMC10948526; doi:10.1007/s10549-023-07147-z)
Supplement: Supplementary file 1 — Supplementary file1 (DOCX 118 KB) [file 10549_2023_7147_MOESM1_ESM.docx]

*Breast Cancer Research and Treatment*

**Radium-223 in women with hormone receptor-positive bone-metastatic breast cancer receiving endocrine therapy: pooled analysis of two international, phase 2, randomized, double-blind, placebo-controlled trials**

Hope S. Rugo^1^, Catherine H. Van Poznak^2^, Patrick Neven^3^, Iwona Danielewicz^4^, Soo Chin Lee^5^, Mario Campone^6^, Jeannie Y. K. Chik^7^, Estela Vega Alonso^8^, Bjørn Naume^9^, Etienne Brain^10^, Jonathan M. Siegel^11^, Rui Li^11^, Deise Uema^11^, Volker J. Wagner^12^, Robert E. Coleman^13^

^1^University of California San Francisco Helen Diller Family Comprehensive Cancer Center, San Francisco, CA, USA; ^2^University of Michigan Rogel Cancer Center, Ann Arbor, MI, USA; ^3^University Hospitals Leuven, Leuven, Belgium; ^4^Szpital Morski im. PCK, Gdynia, Poland; ^5^National University Hospital (S) Pte Ltd, Singapore; ^6^Institut de Cancerologie de l’Ouest, St Herblain, France; ^7^Queen Elizabeth Hospital, Kowloon, Hong Kong; ^8^Centro Integral Oncológico Clara Campal, Madrid, Spain; ^9^Institute of Clinical Medicine, University of Oslo, and Oslo University Hospital, Oslo, Norway; ^10^Institut Curie – René-Huguenin Hospital, Saint-Cloud, France; ^11^Bayer HealthCare Pharmaceuticals Inc., Whippany, NJ, USA; ^12^Bayer Consumer Care AG, Basel, Switzerland; ^13^University of Sheffield, Cancer Clinical Trials Centre, Weston Park Hospital, Sheffield, UK

Corresponding authors:

Hope S. Rugo, [hope.rugo@ucsf.edu](mailto:hope.rugo@ucsf.edu)

Robert E. Coleman, [r.e.coleman@sheffield.ac.uk](mailto:r.e.coleman@sheffield.ac.uk)

**SUPPLEMENTARY MATERIALS**

**Contents**

[Supplementary methods 3](#_Toc131600503)

[Inclusion criteria 3](#_Toc131600504)

[Exclusion criteria 7](#_Toc131600505)

[Study interventions 9](#_Toc131600506)

[Safety assessments 13](#_Toc131600507)

[Randomization 17](#_Toc131600508)

[Blinding 18](#_Toc131600509)

[Study termination 18](#_Toc131600510)

[Suppl Table 1 Demographic and baseline characteristics: Study A 20](#_Toc131600511)

[Suppl Table 2 Demographic and baseline characteristics: Study B 21](#_Toc131600512)

[Suppl Table 3 Efficacy outcomes in the individual studies (intention-to-treat populations) 23](#_Toc131600513)

[Suppl Table 4 TEAEs occurring in ≥15% of patients in either study arm (safety analysis set, *n* = 97): Study A 24](#_Toc131600514)

[Suppl Table 5 TEAEs occurring in ≥15% of patients in either study arm (safety analysis set, *n* = 278): Study B 25](#_Toc131600515)

[Suppl Fig. 1 Study flow. ITT, intention to treat 26](#_Toc131600516)

Supplementary methods

Inclusion criteria

***Both studies***

Patients who met all the following criteria were eligible for study inclusion.

1. *Written informed consent*: Patients had to understand and willingly sign the informed consent form before the conduct of any study-specific procedure
2. *Documentation of histologic or cytologic confirmation of estrogen receptor-positive (ER+) and human epidermal growth factor receptor 2 (HER2)-negative adenocarcinoma of the breast*: HER2 status must have been determined by an accredited or Ministry of Health approved laboratory by immunohistochemistry (IHC), fluorescence *in situ* hybridization (FISH), chromogenic *in situ* hybridization (CISH), or another validated *in situ* hybridization (ISH) assay for the detection of *HER2* gene expression
3. *ER+ tumors*: defined as ≥10% positive tumor nuclei in the analyzed sample from either the primary or a metastatic site; ER+/progesterone receptor-positive (PR+) and ER+/progesterone receptor-negative (PR−) patients were eligible for study inclusion; patients with estrogen receptor-negative (ER−)/PR+ or ER−/PR− disease were ineligible
4. *Women, aged ≥18 years, with metastatic breast cancer not amenable to curative treatment by surgery or radiotherapy*: Women of reproductive potential and their male partners agreed to use adequate contraception during treatment and for 6 months following the completion of radium-223 or placebo administration
5. *Premenopausal or postmenopausal patients*: In premenopausal patients, ovarian radiation or treatment with a luteinizing hormone-releasing hormone agonist/antagonist was permitted for induction of ovarian suppression if the plasma/serum estradiol assay was within the local laboratory premenopausal range at screening, performed within 7 days of randomization
   1. Premenopausal patients with or without ovarian radiation or concomitant treatment with a luteinizing hormone-releasing hormone agonist/antagonist had a negative pregnancy test at screening and agreed to use an adequate method of contraception, as recommended by their treating physicians
   2. Postmenopausal status was defined by either:
      1. Age ≥55 years and ≥1 year of amenorrhea or
      2. Age <55 years and ≥1 year of amenorrhea, with a plasma/serum estradiol assay within the local laboratory postmenopausal range, performed within 7 days of randomization or
      3. Bilateral ovariectomy
6. *Bone-dominant disease*: At least two skeletal metastases identified at baseline by bone scintigraphy and confirmed by computed tomography (CT)/magnetic resonance imaging (MRI); metastases in soft tissue (skin, subcutaneous tissue, muscle, fat, lymph nodes) and/or visceral metastases were permitted
7. *Measurable disease or non-measurable but radiologically evaluable disease, according to Response Evaluation Criteria in Solid Tumors (RECIST) version 1.1*: All disease burden was assessed at baseline by CT or MRI of the chest, pelvis, abdomen, and any additional fields as needed; all patients also had to have a bone scan at baseline
   1. CT/MRI and bone scans done as standard practice within 3 weeks before randomization were acceptable
   2. Fluorodeoxyglucose (FDG)-positron emission tomography (PET), if performed as part of standard-of-care imaging, could be used as an adjunct to CT/MRI, in line with RECIST version 1.1 criteria; if FDG-PET/CT was conducted, the CT component of the scan could be used for tumor measurements only if the study site could document that the CT scan was of identical diagnostic quality to that of diagnostic CT
   3. FDG-PET/CT or ^18^F-sodium fluoride PET/CT was acceptable as an alternative to technetium-99m bone scintigraphy if it was the standard of care at the institution and provided that the same bone-imaging modality was used throughout the study
8. *At least one line of prior hormonal therapy in the metastatic setting*: Any change of the hormone agent because of investigator-assessed progression was counted as a new line of therapy; a switch of hormone therapy from one agent to another due to toxicity or other reasons (e.g., patient preference), in the absence of progressive disease at the time of the switch, was counted as one line, even though two different agents were administered
9. *One or two prior skeletal-related events (SREs)*: defined as a need for external-beam radiation therapy (EBRT) to bone, pathologic bone fracture (excluding major trauma), spinal cord compression, or orthopedic surgery; patients with no prior SREs or more than two prior SREs were excluded
   1. All prior SRE-related procedures had to have been administered before randomization
   2. SREs that occurred ≥21 days apart were defined as separate events, to ensure that linked events (e.g., surgery for fracture repair or multiple doses of radiation during a course of treatment) were not counted as separate events; bone pain that occurred at several anatomical locations and required separate EBRT sessions was counted as one event if the EBRT sessions were administered within 21 days of each other
10. *Bone health agents:* Bisphosphonate or denosumab treatment for ≥1 month before the start of study treatment
11. *Eastern* *Cooperative* *Oncology* *Group* *(ECOG)* *performance* *status* *(PS)* *0 or* *1*
12. *Life* *expectancy* *≥6* *months*
13. *Laboratory requirements*:
    1. Absolute neutrophil count (ANC) ≥1.5 × 10^9^/l
    2. Platelet count ≥100 × 10^9^/l, without platelet transfusion within 4 weeks before randomization
    3. Hemoglobin (Hb) ≥9.0 g/dL (90 g/l; 5.6 mmol/l) without transfusion or erythropoietin within 4 weeks before randomization
    4. Total bilirubin level ≤1.5 × institutional upper limit of normal (ULN), except for patients with documented Gilbert disease
    5. Aspartate aminotransferase (AST) and alanine aminotransferase (ALT) ≤2.5 × institutional ULN
    6. Creatinine ≤1.5 × ULN
       1. Estimated glomerular filtration rate ≥30 ml/min/1.73 m^2^, according to the Modification of Diet in Renal Disease-abbreviated formula
    7. International normalized ratio (INR) of prothrombin time (PT), partial thromboplastin time (PTT), or activated PTT ≤1.5 × ULN at study entry
       1. Patients treated with warfarin, heparin, enoxaparin, rivaroxaban, dabigatran, apixaban, or aspirin (e.g., ≤100 mg daily) could participate in the study if no underlying abnormality in coagulation parameters was known; weekly evaluation of INR/PTT was required until stability was achieved for anticoagulants that required monitoring according to local labels
    8. Serum albumin >30 g/l
    9. Pulse oximetry O_2_ saturation >92%, if lung metastases were present
14. *Ability to swallow oral* *medication*

***Study A-specific inclusion criterion***

1. *Eligibility for further standard-of-care endocrine therapy* with any of the following, administered as a second or later line of hormone therapy in the metastatic setting:
   1. Selective ER modulators, such as tamoxifen or toremifene
   2. Nonsteroidal aromatase inhibitors, such as anastrozole or letrozole
   3. Steroidal aromatase inhibitors, such as exemestane
   4. ER downregulators, such as fulvestrant

Patients started treatment with the single hormone agent either within 15 days before randomization or after randomization, but before or simultaneously with the first injection of radium-223 or placebo

***Study B-specific inclusion criteria***

1. *Recurrent or progressive disease following treatment* with a nonsteroidal aromatase inhibitor (anastrozole or letrozole) in an adjuvant or metastatic setting
2. *Eligibility, on the investigator’s assessment and according to the local label, for treatment with exemestane and everolimus* as a second or later line of hormone therapy in a metastatic setting

Exclusion criteria

***Both studies***

Patients who met any of the following criteria were not eligible to participate in the studies.

1. *HER2-positive breast cancer*: IHC 3+, positive FISH/CISH/other ISH-validated assay; equivocal or unknown HER2 status
   1. Patients with IHC 3+ could not be chosen, regardless of their FISH/CISH/other ISH-validated assay status; patients with positive FISH/CISH/other ISH-validated assay status also could not be chosen, irrespective of the IHC findings; patients with IHC 2+ were ineligible if no negative FISH/CISH/other ISH-validated assay for detection of *HER2* gene expression was available
2. *Everolimus therapy*: Candidates for everolimus as the current intervention for their metastatic breast cancer, in the opinion of the treating investigator (Study A) or prior or current everolimus therapy (Study B)
3. *Inflammatory breast cancer*
4. *Chemotherapy for metastatic disease*: prior chemotherapy or, in the opinion of the treating investigator, appropriate candidates for chemotherapy as the current intervention for their metastatic breast cancer; chemotherapy administered for adjuvant/neoadjuvant disease was acceptable
5. *Previous untreated or concurrent cancer* that was distinct in the primary site or histology from the cancer under study, except treated basal cell carcinoma or superficial bladder tumor (Ta and Tis, American Joint Committee on Cancer, 7th edition). Patients surviving a cancer that was curatively treated and without evidence of disease for >3 years before enrollment could be included. All cancer treatments must have been completed ≥3 years before study entry [i.e., signature date of the informed consent form (ICF)]
6. *Patients with known, or a history of, brain metastases or leptomeningeal disease*: Patients with neurologic symptoms had to have contrast CT/MRI of the brain within 28 days before randomization to exclude active brain metastases; imaging of the central nervous system was otherwise not required
7. *Imminent or established untreated spinal cord compression*, based on clinical findings and/or MRI; following treatment of spinal cord compression, patients could be eligible for inclusion if all other eligibility criteria were satisfied
8. *Prior treatment with radium-223*
9. *Prior hemibody external radiotherapy*: Patients who received other types of prior external radiotherapy could be included, if bone marrow function was assessed and met the protocol requirements for Hb, ANC, and platelets
10. *Prior systemic radiotherapy* with strontium-89, samarium-153, rhenium-186, or rhenium-188
11. *Eastern Cooperative Oncology Group performance status (ECOG PS)* *≥2*
12. *Blood transfusions, platelet transfusions, or use of erythropoietin* within 4 weeks before randomization
13. *Use of biologic response modifiers*, such as granulocyte macrophage-colony stimulating factor or granulocyte-colony stimulating factor, within 4 weeks before randomization
14. *Administration of an investigational drug or any anticancer treatment* within 4 weeks before randomization
15. *Chronic conditions associated with non-malignant abnormal bone growth* (e.g., confirmed Paget’s disease of bone)
16. *Any other serious illness or medical condition,* such as, but not limited to:
    1. Any uncontrolled infection
    2. New York Heart Association class III or IV cardiac failure
    3. Crohn’s disease or ulcerative colitis
    4. Bone marrow dysplasia
17. *Previous assignment to treatment in this study*
18. *Breastfeeding*
19. *Hypersensitivity* to any substance used in this study (e.g., radium-223, exemestane, everolimus, or other rapamycin derivatives) or its excipients
20. *Osteonecrosis of the jaw*
21. *Immediately life-threatening visceral disease*, for which chemotherapy was the preferred treatment option
22. *Lymphangitic* *carcinomatosis*
23. *Ascites requiring paracentesis* within 2 weeks before study entry (i.e., signature date of the ICF) or during the screening period

All local-label-specific criteria for the standard-of-care hormonal treatment, bisphosphonates, and denosumab applied. Patients were treated according to the local standard-of-care requirements.

Study interventions

***Both studies***

All study drugs were labeled according to the requirements of local law and legislation. Label text was approved according to the sponsor’s agreed procedures, and a copy of the labels was made available to the study site upon request. For all study drugs provided by the sponsor, a system of numbering in accordance with all requirements of Good Manufacturing Practice was used, ensuring that each dose of study drug could be traced back to the respective bulk source of the ingredients. Lists linking all numbering levels were maintained by the sponsor’s clinical supplies quality assurance group. For all sponsor-supplied study drugs, lists linking all numbering levels were maintained by the sponsor.

*Radium-223 dichloride*

The alpha particle-emitting radiopharmaceutical radium-223, manufactured by Bayer HealthCare, was a sterile, non-pyrogenic, clear, and colorless aqueous solution of radium-223 dichloride for intravenous administration. Radium-223 dichloride was produced according to Good Manufacturing Practice and was delivered in a glass vial, ready to use, and with a certified activity. Radium-223 dichloride had a physical half-life of 11.4 days. The product was isotonic and had a pH of 6.0–8.0. The radioactive concentration at the reference date was 1100 kBq/ml. The product had a precalibration of 14 days. When administered on a day other than the reference day, the volume was corrected according to the physical decay table supplied with each shipment. The radiopharmaceutical was shipped in a lead container as a Type A radioactive package, according to international transportation guidelines for radioactive materials. The volume per vial was 6 ml, corresponding to 6 MBq at the calibration reference day. Radium-223 dichloride had a shelf life of 28 days from the production day when stored at ambient temperature. The shelf life was demonstrated for temperatures from cold storage (2–8°C) up to 40°C; product quality was not jeopardized by freezing. Study sites in the USA could have a patient-ready dose (PRD) prepared by a radiopharmacy (Cardinal Health), with doses delivered to the study sites in prefilled syringes. Unless otherwise agreed, in cases where study drug had been ordered, the time window for administration was within 3 days of the planned treatment day. If administration was postponed for >3 days, replacement of the drug order was required.

Drug administration

Written information about radium-223 and instructions about handling and injection of radioactive material were provided to study personnel. In general, the administration of radioactive drugs involves a potential risk for third parties, due to radiation from patients and possible contamination from spilled urine or feces. When radium-223 was injected intravenously into a patient, the risk of external radiation exposure to third parties was extremely low, due to the short range of the alpha particles (<100 μm) and the low portion of beta and gamma radiation. Thus, the product could be administered on an outpatient basis. To minimize the risk of contamination, patients and caregivers received oral and written instructions regarding hygiene precautions following administration of a radioactive drug, according to the investigational study site radiation protection guidelines. These instructions were given to all patients, as neither the principal investigator nor the patients knew the assignment to radium­223 or placebo injections.

The study medication was administered as a slow bolus intravenous injection. The actual radioactivity administered was within the tolerance limits of ±10% of the calculated radioactivity. Every effort was made to administer the full dosing regimen, and single dose level adjustments of radium-223 or placebo were not permitted. After administration, the equipment used in connection with preparation and administration of the study drug was treated as radioactive waste and disposed of in accordance with hospital procedure for the handling of radioactive material and according to local laws. Written information about radium-223 dichloride and instructions for the handling and injection of radioactive materials were provided to study personnel.

Dose calibration

Radium-223 was measured in a normal dose calibrator instrument. When all required written approvals for the use and handling of radium-223 from the Radiation Protection Agency for the specific site had been received by the sponsor, a vial of radium-223 dichloride for technical use was sent to the study site. Different clinical study sites have dose calibrators from different suppliers; thus, the isotope calibration factor differed from site to site. Consequently, each site performed the radium-223 dial setting on their relevant dose calibrators if no isotope calibration factor for radium-223 was provided by the vendor of the dose calibrator. For dial setting, the clinical study site received a sealed vial or a prefilled syringe containing a radium-223 solution for calibration only. The vial or syringe was identical to the vials/syringes used for study treatment. The amount of radium-223 in the vial/syringe was stated on the label. Instructions for the dial setting, including the calibration log form, were enclosed with the dispatch of the calibration sample.

Dose handling

At least two unblinded study personnel were nominated at each study site. The primary dedicated unblinded person (“the unblinded person”), who had the responsibility delegated from the principal investigator, was responsible for the safe handling and storage of radium-223 dichloride and placebo control. The unblinded person was also responsible for correctly receiving and recording the delivery of radium-223 dichloride. At least one deputy unblinded person was also nominated. Radium-223 was handled by individuals who were qualified by training and experience in the safe handling of radionuclides. The radium-223 vials or PRDs were stored inside their lead containers in a secure facility. The study drug was used within 28 days of production or before the expiry date specified for PRDs. Control measurements of both the radium-223 dichloride vial (before and after dispensing) and syringes (before and after administration) were performed as part of the clinical study documentation. Because PRDs were prepared at the country depot, relevant procedures were recorded by the country depot staff. All administrations of radium-223 dichloride were based on the certified activity of radium-223 at the reference date. All documentation that contained unblinded information was kept by the unblinded persons and not shared with the other study site personnel during the conduct of the study.

Dose calculation

The dosage of radium-223 dichloride was 55 kBq/kg body weight. The total activity to be injected was calculated volumetrically using the patient’s body weight (kg) within 5 days of injection, the 55 kBq/kg dosage level, and the decay correction factor (DK) to correct for physical decay of radium-223. A table with DKs according to physical decay of the study medication was provided with each vial of radium-223 dichloride. The total amount to be administered to a patient (volume to be drawn into the syringe) was calculated according to the recommended formula:

$$Volume to be injected \left( mL \right)=\frac{\left[ Bodyweight \left( kg \right) \times55 kBq/kg \right]}{\left[ DK \times1100 kBq/mL \right]}$$

Site-specific volume calculation methods were acceptable as well if the patient dose was 55 kBq/kg. Data regarding activity, calculations, and volume to be injected were recorded in the investigational medicinal product (IMP) preparation log and in the study electronic data capture tool (Rave; Medidata, New York, NY, USA) by the unblinded person. This requirement applied both to doses prepared at the study site and to doses prepared by off-site vendors.

Dose preparation

To keep the treating physician blinded to the assignment of study medication, the unblinded person (e.g., from the hospital pharmacy or nuclear medicine department) was responsible for calculating the dose and blinding the syringe. Data regarding activity and volume to be injected were recorded in the IMP preparation log and in the appropriate electronic case record form (eCRF), both of which were unavailable to the treating physician. Copies of the vial label and syringe serial number were attached with each entry in the IMP preparation log. Additional written instructions for study drug administration for blinded and unblinded personnel were provided. Personnel used appropriate protective clothing and equipment (laboratory coats, medical gloves/protective glasses) during syringe filling and application to prevent contamination with the radioactive solution and to reduce radiation exposure. Sites adhered to all relevant radiation safety regulations, as prescribed by local authorities administering the site radiation licenses.

Filling of the syringe took place in a safety bench or similar cabinet in the radiopharmacy/nuclear medicine department. The individual responsible for study drug preparation drew the correct volume of study drug into a syringe. The size of the syringe was chosen according to the applied volume to reach the required dosing accuracy. In some regions, a third-party vendor was used to prepare the injections to be used by study sites. Radium-223 dichloride was not diluted or mixed with any solutions. If the vials were stored in a refrigerator, they were left at room temperature for 1 h before use, because cold material was not to be injected into patients. To maintain traceability, each patient was assigned one syringe label set with a unique serial number that was linked to the vial/batch received with the preparation and blinded administration of the syringe.

*Placebo*

Isotonic saline solution (0.9% sodium chloride solution for injection) was provided by the study site. Traceability of the respective manufacturers and batches was maintained in the respective preparation documentation and drug accountability logs. For saline solution provided to patients from the study site’s commercial supply, it was required at minimum that the assignment, batch numbers, and expiry dates were recorded. The use of saline was recorded, and documentation was retained at the study site with a copy provided for the sponsor study file.

The volume of saline solution to be injected was provided by the interactive voice/web response system (IXRS) based on each patient’s body weight. Data regarding the saline batch number and the volume to be injected were recorded in Rave. Each syringe containing isotonic saline solution was prepared in the same way as for the active treatment, including the use of unique serial numbers on the syringe.

*Dose adjustments, delays, and discontinuations*

Radium-223 or placebo administration could be delayed for up to 4 weeks (maximum 8 weeks between two injections) for recovery from adverse events (AEs). If administration had to be delayed for >4 weeks, radium-223 or placebo administration was discontinued. AEs were reported and graded according to National Cancer Institute Common Terminology Criteria for AEs (NCI-CTCAE) version 4.03.

Safety assessments

All patients who received at least one dose of study drug were valid for safety analysis. All AEs were reported, and severity graded, using NCI-CTCAE version 4.03. The following safety variables were evaluated:

- AEs recorded on an ongoing basis throughout the study
  - An AE was any untoward medical occurrence [i.e., any unfavorable and unintended sign (including abnormal laboratory findings), symptom, or disease] in a patient after providing written informed consent for study participation; therefore, an AE may or may not be temporally or causally associated with the use of a medicinal (investigational) product. A surgical procedure that was planned before the start of the study by any physician treating the patient was not recorded as an AE (however, the condition for which the surgery was required was considered to be an AE)
  - A serious AE (SAE) was classified as any untoward medical occurrence that, at any dose, met any of the following criteria:
    - Resulted in death
    - Was life-threatening; the term “life-threatening” referred to an event for which the patient was at risk of death at the time of the event; it did not refer to an event that, if more severe, might have caused death
    - Required inpatient hospitalization or prolongation of existing hospitalization. Such hospitalization was not regarded as an SAE if at least one of the following exceptions were met: the admission resulted in a hospital stay of <12 h; the admission was preplanned (i.e., elective or scheduled surgery arranged before study start); the admission was not associated with an AE (e.g., social hospitalization for purposes of respite care). However, invasive treatment during any hospitalization fulfilled the criterion of “medically important” and, as such, was reportable as an SAE, dependent on clinical judgment. In addition, where local regulatory authorities specifically required a more stringent definition, the local regulation took precedence
    - Resulted in persistent or significant disability or incapacity; disability meant a substantial disruption of a person’s ability to conduct normal life functions
    - Was a congenital anomaly or birth defect
    - Was another medically important serious event, as judged by the investigator
    - Was an occurrence of any additional malignancies, including acute myeloid leukemia, or hematologic conditions, such as myelodysplastic syndrome, aplastic anemia, or myelofibrosis (regardless of the investigator’s causality assessment). If disease progression led to signs and symptoms that met the criteria for seriousness (e.g., hospitalization), the associated signs and symptoms, not the underlying cause, were reported as SAEs (i.e., “progressive disease” was not recorded as an SAE); in this case, disease progression was mentioned on the SAE form as an “alternative explanation”
  - Any isolated laboratory abnormality that met the criteria for NCI-CTCAE grade 4 classification was not reportable as an SAE unless the investigator assessed that the event met standard International Conference on Harmonization criteria for an SAE; all laboratory abnormalities, including NCI-CTCAE grade 4 abnormalities, were documented on the laboratory eCRF (including values reported from local laboratories)
- Causal relationship of AE to study drug
  - Assessment of the causal relationship between an AE and treatment administration was a clinical decision based on all available information when the eCRF was completed; the assessment was based on whether there was a “reasonable causal relationship” to the study treatment in question; potential causal relationships of the backbone hormone treatment, bisphosphonates, denosumab, or other concomitant medication were also collected
- Safety variables included the analysis of acute and long-term effects; the appearance of new primary malignancies; hematopoietic reserve for tolerability of subsequent chemotherapy; and bone fractures and bone-associated events, including during long-term follow-up, regardless of the investigator’s causality assessment; other safety variables included vital signs (blood pressure, body weight, heart rate, respiratory rate, and body temperature), physical examination findings, electrocardiogram data, and ECOG PS score
- Laboratory assessments:
  - Hematology: hematocrit, Hb, platelet count, red blood cell count, white blood cell count, and white blood cell differential
  - Clinical chemistry: sodium, potassium, chloride, calcium, total cholesterol, ALT, AST, lactate dehydrogenase, bone ALP, serum creatinine, phosphate, blood urea nitrogen, total bilirubin, and albumin
  - A coagulation panel: PT, PTT, and INR

***Documentation and assessment of AEs***

All AEs that occurred from the time when the patient signed the ICF up to 30 days after the last dose of study medication were recorded on the eCRF. Treatment-emergent AEs (TEAEs) and all SAEs that occurred during the treatment period and up to 30 days after the last administration of any study medication (radium-223/placebo, exemestane, or everolimus) were reported on the appropriate eCRF. All SAEs that occurred during the treatment period and up to 30 days after the last administration of study medication were reported immediately (within 24 h of the investigator’s awareness) in the appropriate eCRF. If more than one AE occurred, each event was recorded separately. All AEs and SAEs were followed until resolution, or as clinically required. All AEs and SAEs occurring beyond 30 days after the last dose of study treatment were documented and reported, if considered related to study medication or to study-related procedures.

Patients who received cytotoxic chemotherapy during the follow-up period were observed for the development of febrile neutropenia and hemorrhage during and for up to 6 months after chemotherapy. Occurrence of these AEs was documented and reported if considered related to the chemotherapy. All occurrences of additional malignancies, including acute myeloid leukemia, and hematologic conditions, such as myelodysplastic syndrome, aplastic anemia, and myelofibrosis, were reported as SAEs, regardless of the investigator’s assessment.

Relevant symptoms related to symptomatic skeletal events were captured as AEs or SAEs as appropriate, regardless of the relationship to study drug, from the date of first dose until the end of active follow-up. All bone fractures and bone-associated events (e.g., osteoporosis) were collected as either AEs or SAEs as appropriate, regardless of the investigator’s causality assessment.

AEs were reported spontaneously by the patient or elicited through open (non-leading) questioning during each visit to the clinic, and at completion of the active follow-up period without clinic visits. As far as possible, all AEs were described by their duration (start and stop date), severity (graded according to the NCI-CTCAE version 4.03), relationship to treatment, and the need for other specific therapy. All information was recorded in the source documentation and eCRF.

Laboratory test abnormalities considered clinically relevant (e.g., abnormalities that caused the patient to withdraw from the study), that required treatment, that caused clinical manifestations, or that were judged as relevant by the investigator were reported as an AE. Each event was described in detail, with start and stop dates (onset and resolution of event), severity, temporal and causal relationship to the investigational product and/or protocol-related procedures, potential other factors (e.g., comorbidities, comedications), therapeutic action taken, result of the therapeutic action, and ultimate outcome of the AE. The investigator’s assessments of AEs and laboratory results with grades and causality assessments were documented and retained in the source documentation. If more than one AE occurred, each event was recorded separately. All AEs and SAEs were followed until resolution, or as clinically required.

Randomization

For random assignment of radium-223 or placebo administration, a computer-generated randomization list was prepared by the sponsor and provided to the IXRS. The IXRS assigned each eligible patient a randomization number and the respective treatment in a ratio of 1:1 to radium-223 or placebo. Randomization was stratified by study ID in the pooled analysis; by geographic region [Europe (including Israel) and North America versus Asia] and previous lines of endocrine therapy in the metastatic setting (one versus two or more) in both studies; and by prior SREs (one versus two) in Study A or visceral metastases (present versus absent) in Study B. The IXRS provided the randomization number but not the assigned treatment to the caller (i.e., blinded personnel). A confirmation e-mail containing the randomization number was sent to unblinded personnel, who then logged into the IXRS to discover the treatment arm assigned to the patient. Unblinded personnel were responsible for preparing the study drug for the patient for the first administration. In the USA only, if a patient was allocated to radium-223, an unblinded person faxed the shipment request to Cardinal Health for a prefilled syringe. The timing for the drug order was based on the planned patient visit date. If the patient was allocated to placebo, the unblinded person at the study site was responsible for providing saline solution corresponding to the IXRS treatment day. This information was not made available before the IXRS treatment day, to avoid unblinding of the patient and blinded study personnel. Subsequent orders for each study drug administration were made by a call to the IXRS from the study site on the date of the previous injection. If, after ordering, the order needed to be canceled or amended, the investigator contacted their monitor immediately.

Blinding

Every effort was made to maintain study blinding. Patients were randomized to receive radium­223 or placebo in a double-blind fashion. All patients also received appropriate backbone standard-of-care hormonal therapy, which was locally sourced by the site and was provided in an open-label format. Because of the nature of radium-223, two or more people in each study site’s nuclear medicine department were unblinded to the treatment assigned to patients. One of these unblinded individuals served as a back-up for the other. To maintain the study blind for hospital personnel who provided treatment to each patient, the unblinded person at the study site was responsible for filling the syringe with the correct amount of radium-223 or placebo (saline solution) and labeling it. Both radium-223 and placebo were clear solutions; thus, syringes with radium-223 and placebo could not be visually distinguished. The person performing the study drug administration was blinded to the treatment allocation. Patients were not told whether they had received radium-223 or placebo. All treating physicians, clinical staff, patients, and sponsor personnel were blinded to the treatment to which a patient was randomized, except for named representatives who performed the verification of drug accountability at study sites and drug ordering.

Study termination

An administrative interim analysis of efficacy and safety was conducted on December 5, 2017 to inform phase 3 development plans for radium-223 in breast cancer. The interim data review was added by Protocol Amendment 7 of Study A, to be performed when approximately 40 radiological progression-free survival (rPFS) events had occurred. The interim data review was added by Protocol Amendment 8 of Study B, to be performed when a minimum of 80 rPFS events had been reached. Enrollment was halted while this review was undertaken.

An independent team of statisticians generated unblinded statistical outputs based on pooled data from Study A and Study B, which were reviewed by an internal data review committee (IDRC), independent of the study team. The IDRC decision on whether to proceed to phase 3 was based on predefined efficacy criteria laid out in its operational plan and not based on a safety signal. As prespecified in the IDRC operational plan, statistical testing was performed for symptomatic skeletal event-free survival (primary endpoint) and rPFS. Because the review was not intended to assess futility or superiority of the individual studies, no alpha adjustment, beta spending, or power adjustment was applied for this interim analysis. However, after the IDRC review, as a result of slow recruitment due to changes in the treatment landscape, enrollment was not continued in either study.

Suppl Table 1 Demographic and baseline characteristics: Study A

| **Patient demographics and baseline characteristics (intention-to-treat population, *N* = 99)** | **Radium-223 + endocrine therapy (*n* = 49)** | **Placebo + endocrine therapy (*n* = 50)** |
| --- | --- | --- |
| Age at baseline, mean (SD), years | 57.1 (11.5) | 58.7 (12.0) |
| Age group, *n* (%) |  |  |
| <55 years | 19 (38.8) | 19 (38.0) |
| ≥55 years | 30 (61.2) | 31 (62.0) |
| ECOG performance status at baseline, *n* (%) |  |  |
| 0 | 25 (51.0) | 20 (40.0) |
| 1 | 24 (49.0) | 30 (60.0) |
| Menopausal status at baseline, *n* (%) |  |  |
| Premenopausal | 7 (14.3) | 4 (8.0) |
| Postmenopausal | 42 (85.7) | 46 (92.0) |
| Progesterone receptor status at initial diagnosis, *n* (%) |  |  |
| Positive | 43 (87.8) | 36 (72.0) |
| Negative | 4 (8.2) | 10 (20.0) |
| Unknown | 2 (4.1) | 4 (8.0) |
| Previous lines of endocrine therapy in metastatic setting, *n* (%) |  |  |
| 1 | 32 (65.3) | 32 (64.0) |
| ≥2 | 17 (34.7) | 18 (36.0) |
| Metastatic status at baseline, *n* (%) |  |  |
| Bone metastases only | 35 (71.4) | 30 (60.0) |
| Bone plus visceral metastases | 6 (12.2) | 9 (18.0) |
| Bone plus non-visceral metastases | 8 (16.3) | 11 (22.0) |
| Prior SREs, *n* (%) |  |  |
| 1 | 34 (69.4) | 31 (62.0) |
| ≥2 | 15 (30.6) | 19 (38.0) |

ECOG, Eastern Cooperative Oncology Group; SD, standard deviation; SRE, skeletal-related event.

Suppl Table 2 Demographic and baseline characteristics: Study B

| **Patient demographics and baseline characteristics**  **(intention-to-treat population, *N* = 283)** | **Radium-223 + endocrine- based therapy (*n* = 142)** | **Placebo + endocrine-based therapy (*n* = 141)** |
| --- | --- | --- |
| Age at baseline, mean (SD), years | 60.0 (10.4) | 59.1 (11.6) |
| Age group, *n* (%) |  |  |
| <55 years | 44 (31.0) | 51 (36.2) |
| ≥55 years | 98 (69.0) | 90 (63.8) |
| ECOG performance status at baseline, *n* (%) |  |  |
| Missing data | 0 | 3 (2.1) |
| 0 | 64 (45.1) | 69 (48.9) |
| 1 | 78 (54.9) | 69 (48.9) |
| Menopausal status at baseline, *n* (%) |  |  |
| Premenopausal | 10 (7.0) | 14 (9.9) |
| Postmenopausal | 132 (93.0) | 127 (90.1) |
| Progesterone receptor status at initial diagnosis; *n* (%) |  |  |
| Positive | 117 (82.4) | 105 (74.5) |
| Negative | 22 (15.5) | 30 (21.3) |
| Unknown | 3 (2.1) | 6 (4.3) |
| Previous lines of endocrine therapy in metastatic setting, *n* (%) |  |  |
| 1 | 75 (52.8) | 75 (53.2) |
| ≥2 | 67 (47.2) | 66 (46.8) |
| Metastatic status at baseline, *n* (%) |  |  |
| Bone metastases only | 55 (38.7) | 59 (41.8) |
| Bone plus visceral metastases | 66 (46.5) | 67 (47.5) |
| Bone plus non-visceral metastases | 21 (14.8) | 15 (10.6) |
| Prior SREs, *n* (%) |  |  |
| Missing data | 0 | 2 (1.4) |
| 1 | 96 (67.6) | 94 (66.7) |
| 2 | 46 (32.4) | 45 (31.9) |

ECOG, Eastern Cooperative Oncology Group; SD, standard deviation; SRE, skeletal-related event.

Suppl Table 3 Efficacy outcomes in the individual studies (intention-to-treat populations)

| Outcome | Study A | | Study B | |
| --- | --- | --- | --- | --- |
|  | Radium-223  (*n =* 49) | Placebo  (*n* = 50) | Radium-223  (*n =* 142) | Placebo  (*n =* 141) |
| Symptomatic skeletal event-free survival |  |  |  |  |
| Events, *n* (%) | 21 (42.9) | 26 (52.0) | 69 (48.6) | 80 (56.7) |
| Median (80% CI), months | 30.1 (21.8–43.0) | 18.4 (9.1–28.2) | 21.1 (17.1–23.6) | 19.9 (16.2–24.2) |
| HR (95% CI) | 0.745 (0.409–1.356) | | 0.891 (0.643–1.233) | |
| Radiological progression-free survival |  |  |  |  |
| Events, *n* (%) | 40 (81.6) | 39 (78.0) | 103 (72.5) | 106 (75.2) |
| Median (80% CI), months | 8.1 (5.7–10.6) | 5.8 (5.1–7.9) | 7.9 (6.2–9.7) | 6.7 (5.4–8.1) |
| HR (95% CI) | 1.023 (0.640–1.637) | | 0.874 (0.660–1.157) | |
| Overall survival |  |  |  |  |
| Events, *n* (%) | 18 (36.7) | 19 (38.0) | 73 (51.4) | 73 (51.8) |
| Median (80% CI), months | 43.0 (22.9–NE) | 32.4 (23.7–NE) | 25.0 (23.0–31.4) | 26.4 (21.7–28.9) |
| HR (95% CI) | 0.888 (0.458–1.724) | | 0.968 (0.697–1.343) | |
| Time to bone alkaline phosphatase progression |  |  |  |  |
| Events, *n* (%) | 6 (12.2) | 10 (20.0) | 29 (20.4) | 35 (24.8) |
| Median (80% CI), months | 10.2 (8.3–NE) | 4.3 (3.7–8.3) | 6.1 (4.4–8.7) | 4.3 (3.4–6.1) |
| HR (95% CI) | 0.583 (0.201–1.686) | | 0.645 (0.374–1.114) | |

CI, confidence interval; HR, hazard ratio; NE, not estimable.

Suppl Table 4 TEAEs occurring in ≥15% of patients in either study arm (safety analysis set, *n* = 97): Study A

| TEAE, *n* (%) | Radium-223  (*n =* 48) | Placebo  (*n =* 49) |
| --- | --- | --- |
| Any TEAE | 46 (95.8) | 46 (93.9) |
| Headache | 13 (27.1) | 4 (8.2) |
| Fatigue | 12 (25.0) | 9 (18.4) |
| Nausea | 12 (25.0) | 9 (18.4) |
| Arthralgia | 10 (20.8) | 11 (22.4) |
| Bone pain | 10 (20.8) | 8 (16.3) |
| Back pain | 10 (20.8) | 7 (14.3) |
| Diarrhea | 10 (20.8) | 7 (14.3) |

TEAE, treatment-emergent adverse event (i.e., any event occurring or worsening during radium-223/placebo therapy or up to 30 days after the last administration of radium-223/placebo).

Suppl Table 5 TEAEs occurring in ≥15% of patients in either study arm (safety analysis set, *n* = 278): Study B

| TEAE, n (%) | Radium-223  (*n =* 139) | Placebo  (*n =* 139) |
| --- | --- | --- |
| Any TEAE | 139 (100.0) | 136 (97.8) |
| Stomatitis | 66 (47.5) | 69 (49.6) |
| Anemia | 54 (38.8) | 40 (28.8) |
| Decreased appetite | 48 (34.5) | 34 (24.5) |
| Diarrhea | 43 (30.9) | 31 (22.3) |
| Nausea | 41 (29.5) | 30 (21.6) |
| Fatigue | 38 (27.3) | 41 (29.5) |
| Asthenia | 29 (20.9) | 25 (18.0) |
| Neutropenia | 29 (20.9) | 11 (7.9) |
| Weight decreased | 28 (20.1) | 22 (15.8) |
| Arthralgia | 27 (19.4) | 34 (24.5) |
| Peripheral edema | 26 (18.7) | 25 (18.0) |
| Cough | 25 (18.0) | 26 (18.7) |
| Vomiting | 25 (18.0) | 24 (17.3) |
| Headache | 24 (17.3) | 29 (20.9) |
| Thrombocytopenia | 23 (16.5) | 9 (6.5) |
| Pyrexia | 22 (15.8) | 16 (11.5) |
| Rash | 21 (15.1) | 30 (21.6) |
| Alanine aminotransferase increased | 21 (15.1) | 23 (16.5) |
| Pain in extremity | 21 (15.1) | 19 (13.7) |
| Back pain | 15 (10.8) | 27 (19.4) |
| Bone pain | 12 (8.6) | 21 (15.1) |

TEAE, treatment-emergent adverse event (i.e., any event occurring or worsening during radium-223/placebo, exemestane, or everolimus therapy or up to 30 days after the last administration of radium-223/placebo, exemestane, or everolimus, whichever came last).

Suppl Fig. 1 Study flow. ITT, intention to treat

**
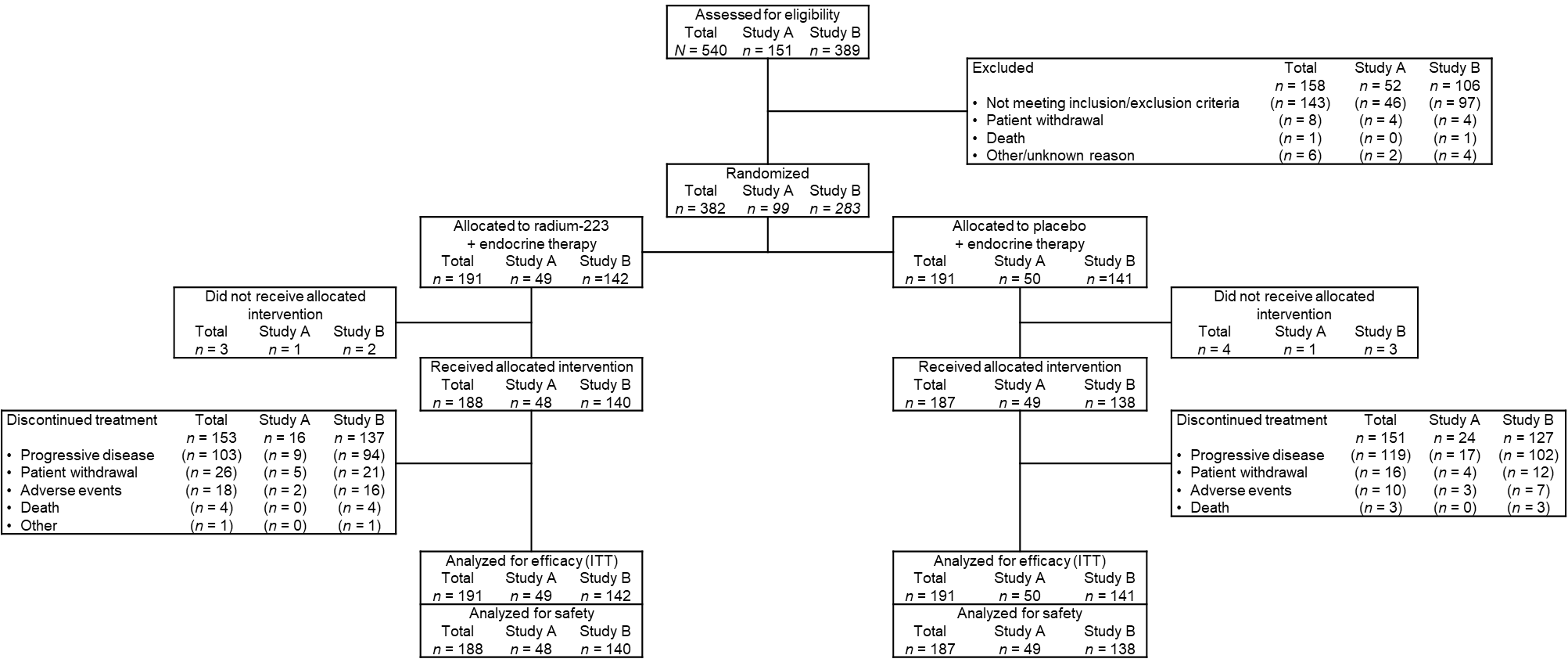
**
